# IL-36 s in the colorectal cancer: is interleukin 36 good or bad for the development of colorectal cancer?

**DOI:** 10.1186/s12885-020-6587-z

**Published:** 2020-02-03

**Authors:** Feier Chen, Meng Qu, Feng Zhang, Zhenyu Tan, Qinghua Xia, Brett D. Hambly, Shisan Bao, Kun Tao

**Affiliations:** 1 Department of Pathology, Tongren Hospital, Shanghai Jiaotong University School of Medicine, Shanghai, China; 2 Discipline of Pathology, School of Medical Sciences and Bosch Institute, Charles Perkins Centre, The University of Sydney, Sydney, Australia; 3 Centre for Disease Control and Prevention of Changning District, Shanghai, China; 4 Beihua University School of Medicine, Jilin, China

**Keywords:** IL36, Colorectal cancer, Prognosis, Multivariate analysis

## Abstract

**Background and aims:**

Colorectal cancer (CRC) is a major killer. Host immunity is important in tumorigenesis. Direct comparison among IL-36α, IL-36β and IL-36γ in the prognosis of CRC is unclear.

**Methods:**

CRC tissue arrays were generated from colorectostomy samples with TNM stage, invasion depth and the demography of these patients (*n* = 185). Using immunohistochemistry/histopathology, IL-36α, IL-36β and IL-36γ were determined, in comparison to non-cancer tissues.

**Results:**

A significant association was observed between colonic IL-36α, IL-36β or IL-36γ and the presence of cancer (with all *P* < 0.0001). Using ROC curve analysis, specificity and sensitivity of IL-36α, IL-36β or IL-36γ were confirmed, with area under the curve (AUC) values of 0.68, 0.73 and 0.65, respectively. Significant differences in survival were observed between IL-36α^high^ and IL-36α^low^ (*P* = 0.003) or IL-36γ^high^ and IL-36γ^low^ (*P* = 0.03). Survival curves varied significantly when further stratification into sub-groups, on the basis of combined levels of expression of two isotypes of IL-36 was undertaken. A significant difference was observed when levels of IL-36α and IL-36β were combined (*P* = 0.01), or a combination of IL-36α plus IL-36γ (*P* = 0.002). The sub-groups with a combination of IL-36α^high^ plus IL-36β^high^, or IL-36α^high^ plus IL-36γ^low^ exhibited the longest survival time among CRC patients. In contrast, the sub-groups of IL-36α^low^ plus IL-36β^high^ or IL-36α^low^ plus IL-36γ^high^ had the shortest overall survival. Using the log-rank test, IL-36α^high^ expression significantly improved survival in patients with an invasion depth of T4 (*P* < 0.0001), lymph node metastasis (*P* = 0.04), TNM III-IV (*P* = 0.03) or with a right-sided colon tumour (*P* = 0.02). Similarly, IL-36γ^low^ expression was significantly associated with improved survival in patients with no lymph node metastasis (*P* = 0.008), TNM I-II (P = 0.03) or with a left-sided colon tumour (*P* = 0.05). Multivariate analysis demonstrated that among IL-36α, IL-36β and IL-36γ, only IL-36α (HR, 0.37; 95% CI, 0.16–0.87; P = 0.02) was an independent factor in survival, using Cox proportional hazards regression analysis.

**Conclusion:**

IL-36α or IL-36γ are reliable biomarkers in predicting the prognosis of CRC during the later or early stages of the disease, respectively. Combining IL-36α plus IL-36γ appears to more accurately predict the postoperative prognosis of CRC patients. Our data may be useful in the management of CRC.

## Background

Colorectal cancer (CRC) is still the third most common cancer, particularly in Western society, despite decades of extensive clinical and basic research [1]. The incidence in China is also rising, partly due to modernisation and/or industrialisation in food processing, which modifies traditional Chinese food preparations [2]. The largest clinical challenge is the delay in early detection [3], compromising the outcomes of CRC patients who consequently may have to be managed with palliative care [4]. Understanding underlying mechanisms involved in the development of CRC would be beneficial to improve the diagnosis and outcomes of CRC patients.

It is well known that host immunity is critical in the development of cancer(s), for example, the discovery of cancer therapy by inhibition of negative immune regulation [5].

IL-36α, β and γ (formerly IL-1F6, IL-1F8, and IL-1F9) are IL-1 family members that signal through the IL-1 receptor, i.e. IL-1Rrp2 (IL-1RL2) and IL-1RAcP [6], via activating the nuclear factor kappa B (NF-κB), Mitogen-activated protein kinase (MAPKs), Jun N-terminal kinases (JNK), and ERK1/2 kinase cascades [7], which are key signalling pathways for intestinal tumorigenesis [8] [9]. The IL-36 isotypes bind to the IL-36 receptor (IL1RL2/IL-1Rrp2/IL-36 receptor dimer) with varying affinities. IL-36 agonists induce various proinflammatory mediators [6] via activating NF-κB and MAPKs. IL-36 is derived from keratinocytes, human monocytes and myeloid dendritic cells (DCs) [10]. IL-36 plays an important role in autoimmune diseases, including psoriatic arthritis, systemic lupus erythematosus and Sjogren’s syndrome [11]. In addition, IL-36 is important in the inflammation of colonic mucosa and promotes inflammation during intestinal diseases, suggesting that IL-36 may be an important therapeutic target for the management of intestinal abnormalities [11]. Furthermore, IL-36 gene therapy may mediate a therapeutic effect in a fibrosarcoma mouse model [12].

The relationship between CRC and IL-36α has been reported previously, showing that high colonic production of IL-36α is beneficial for survival of CRC patients [13]. Interestingly, no non-cancer tissue was included for comparison during the investigation of IL-36α by this research team [13]. Additionally Weinstein et al investigated the relationship between IL-36γ and tertiary lymphoid structure and inflammatory immunity in CRC [14], showing that IL-36γ plays a physiological role in the colon, enhancing the development of CRC via inflammation in the tumour microenvironment. However, the relationship between both IL-36β and IL-36γ and clinicopathological factors in CRC has not been examined.

Therefore, it is of great interest to determine whether there is a correlation among these three IL-36 s (IL-36α, IL-36β and IL-36γ) in terms of clinicopathological outcomes in CRC, using univariate and multivariate analysis. In the current study, we substantially increased the number of CRC patients investigated and used objective computerised automated quantification to determine the production of all the colonic IL-36 s. In addition, we have explored the correlation of IL-36α, IL-36β or IL-36γ production in the CRC patients, comparing cancer versus non-cancer tissues, and the relationship between IL-36α, IL-36β and IL-36γ in the CRC patients. Such data may be useful for both basic research as well as for clinical practice.

## Methods

### Demography of CRC patients and samples

Tissue was collected from adenocarcinoma colorectal cancer (CRC) patients who had undergone colorectostomy at Tongren Hospital, Shanghai Jiaotong University School of Medicine from 2013 to 2017 (*n* = 185). Matched non-cancer tissues (*n* = 130) from the adjacent histopathologically normal tissues was also collected (55 matched non-cancer tissue samples were unavailable for processing or analysis for technical reasons) (Table 1). There were 127 cases of colonic cancer and 58 rectal cancer samples. The selection of CRC adenocarcinoma is expected to be sporadic colon cancer which likely involves abnormalities in the APC/WNT pathway [15]. On the other hand, the mucinous adenocarcinoma form of CRC is likely due to mismatch repair gene mutations [16], and were excluded for this reason. Cancer differentiation grades were based on the guideline of the Royal College of Pathology of Australia COLORECTAL CANCER STRUCTURED REPORTING PROTOCOL (3nd Edition 2016) [17].

**Table 1:** Clinicopathological characteristics of patients with CRC

Characteristics	Total	Ascending (*n* = 21)	Transverse (*n* = 6)	Descending (*n* = 48)	Sigmoid (n-50)	Rectal (*n* = 60)
Gender						
Male/ Female	117/68	12/9	1/5	31/17	31/19	42/18
Age (years)						
< 70/ ≥70	92/93	9/12	3/3	22/26	23/27	35/25
Size (diameter, cm)						
≤ 5/ > 5	141/44	15/6	3/3	32/16	40/10	51/9
Lymph node metastasis						
No/ Yes	110/75	15/6	3/3	23/25	29/21	40/20
Differentiation						
Well/ Moderate/ Poor	4/145/36	0/20/1	0/2/4	0/34/14	3/39/8	1/50/9
Invasion depth						
T1/ T2/ T3/ T4	9/26/31/119	1/4/3/13	0/1/0/5	0/5/6/37	3/8/10/29	5/8/12/35
TNM						
I/II/III/IV	31/78/66/10	5/10/5/1	0/2/4/0	4/19/21/4	10/19/20/1	12/28/16/4
Comorbidity						
Hypertension	31	4	0	10	12	5
Coronary artery atherosclerosis	5	0	0	3	2	0
Type II diabetes	11	1	0	7	1	2
Complication						
Intestinal obstruction	26	6	4	9	6	1
Gastrointestinal perforation	6	0	0	3	3	0
Polyps of the colon	5	2	1	1	1	0
Anaemia	5	1	0	4	0	0

No patients in this study received neoadjuvant therapy, since this study was retrospective and neoadjuvant therapy was not part of treatment protocols at that time. Exclusion criteria were the presence or history of colonic inflammatory pathology, specifically inflammatory bowel disease or diverticulitis.

Seventy nine out of 185 CRC patients had follow-up. The most updated information (till May 2018) showed 52 CRC patients were still alive and 27 were dead. Fifty-three months was the longest survival period for these CRC patients. Tissue arrays with matched non-CRC tissues exhibited a range of different levels of differentiation, invasion and metastasis and were generated in the Department of Pathology, Tongren Hospital, as described previously [18].

Right-sided colon cancer (RCC) is derived from the embryologic midgut (corresponding to the arterial territory of the superior mesenteric artery, including the proximal two-thirds of the transverse colon, ascending colon, and cecum. Left-sided colon cancer (LCC) is derived from the embryologic hindgut, corresponding to the arterial territory of the inferior mesenteric artery, which includes the distal third of the transverse colon, splenic flexure, descending colon, sigmoid colon, and upper rectum [19].

The age cut off of 70 years used in the current study was based on the report by Brenner et al. on Colorectal cancer [20], indicating that Median age at diagnosis is about 70 years in developed countries for colorectal cancer. While it has been determined that the median for diagnosis for males and females varies slightly (72 versus 63 for males versus females) we chose the use the average value of 70 years to maintain statistical power.

The median value for IL-36 used in predicting survival of CRC patients was based on the expression from the CRC tissues, but not non-cancer nor combined data, because of the substantially higher levels of IL-36 in the non-cancer tissues.

The tissues within the pathology blocks were obtained from the patents at surgery with oral consent for surgery including diagnostic and research purpose in an unidentified manner. All of the patients were adults who were older than 16 years. Our current experiment has been approved by *the Human Ethic committee of Tongren Hospital, Shanghai Jiaotong University School of Medicine* for the tissues and the associated deidentified clinical data (ZH2018ZDA33).

### Immunohistochemistry

Immunohistochemistry was performed, as described previously [21]. Briefly, small cores from formalin fixed, wax embedded CRC and non-cancer tissue blocks were embedded in a Tissue Microarray (TAM), as described in detail previously [22]. Each CRC or non-cancer block was sampled five times. Sections (4 μm) from TAMs were checked for consistency with previous histopathological evaluation by HE staining, prior to labelled with rabbit anti-human IL-36α (1/1200) (ab180909), rabbit anti-human IL-36β (1/3200) (ab180890) and rat anti-human IL-36γ (1/1200) (ab156783) antibodies (Abcam, Cambridge, UK). Immunohistochemistry was performed as described previously [22]. The sections were further antigen retrieved (EDTA retrieval buffer, pH 9.0) for 10 min at 95 °C following dewaxing and rehydration, and then treated with 3% H_2_O_2_ for 20 min at room temperature. Rabbit serum (1/1000 diluted in PBS) was used for non-specific blocker. Horseradish peroxidase-conjugated secondary antibody (1/2) (Beijing Sequoia Jinqiao Biological Technology) was applied. DAB was used for visualisation of the specific target. Colonic IL-36α, IL-36β and IL-36γ production was quantified objectively using ImagePro Plus 9.1, as described [23] [24].

### Photograph and image analysis

The image analysis was acquired as described previously [22]. Thirty photomicrographs of each of the labelled slide were taken using Olympus BX63 in manual mode. In order to obtain Integrated Option Density (IOD), these photos were measured using a macro in ImagePro Plus 9.1 software (Media Cybernetics, Rockville, MD, USA). Finally, the average of the IOD from each sample was calculated [23] [24]. The Integrated Option Density obtained was more than 10,000 in the figure. To simplify the score, we scaled it down to 0–1 or 0–4 for the relevant graphs.

### Statistical analysis

Statistics were performed as described previously [18, 25], using GraphPad Prism V9. Wilcoxon signed-rank test or Mann-Whitney U was used for comparison between two paired or non-paired groups. The low and high cut-off values for IL-36α, IL-36β and IL-36γ production were defined by the median of the CRC tissue image unit. The overall survival or survival curves was defined or plotted as previously described [18]. Prognostic factors that influenced survival was determined using Cox’s proportional hazards model. The median of IL-36α, β or γ was obtained from 79 CRC patients yielding a result of 39, i.e. anything above or below 39 was classified as high or low expression. Kruskal-Wallis H was used for multiple comparison. Cox was used for univariate and multivariate analysis, as described [18].

## Results

### Demographic information of the patients

The demographic information from these primary CRC patients, that included matched controls, is shown in Table 1. Actual numbers for some comparisons, however, were slightly lower due to the lack of complete clinical data in a small number of CRC patients, e.g. for the left- or right-sided CRC patients the numbers were 131 or 52, respectively. The numbers of patients with well, moderate or poorly differentiated CRC were 4, 145 and 36, respectively, based on the criteria of histological grading of CRC [26]. The number of tumours whose size was smaller or larger than 5 cm was 141 or 44, respectively. Selection of the tumour size cut-off of 5 cm is well recognised as being of prognostic value in CRC [27]. An age cut-off of 70 years was selected based on the study by Brenner et al. that showed that the median age at CRC diagnosis in developed countries is approximately 70 years [20].

### Comparison of IL-36α, IL-36β and IL-36γ between CRC vs non-CRC in the patient cohort

**Fig. 1:**
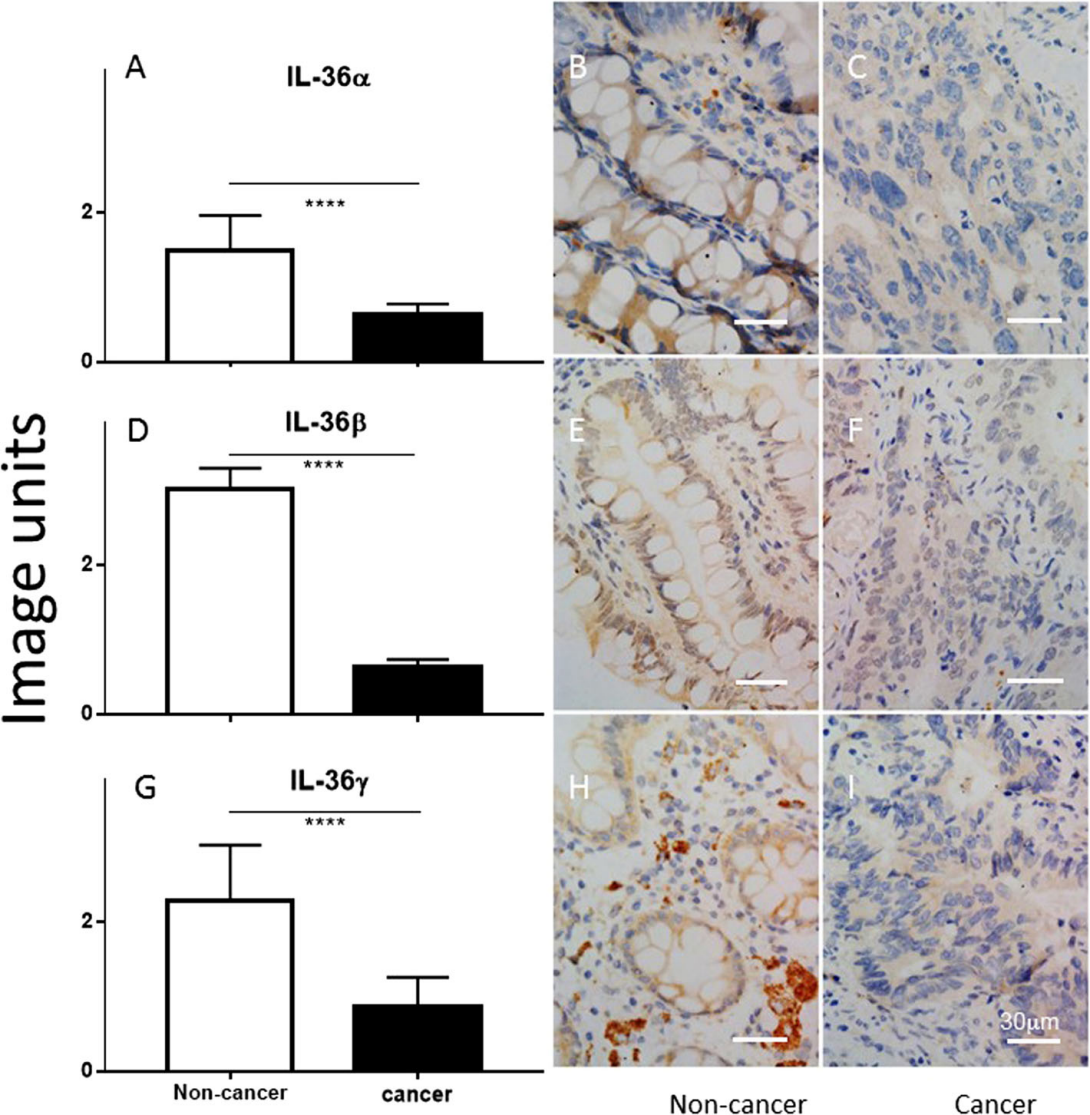
Comparison of the production of IL-36α, IL-36β and IL-36γ between non-cancer colon tissue versus cancer tissue in CRC (**a**, **d** and **g**, respectively), using Wilcoxon signed-rank test. Y-axis represents arbitrary image units. Representative images of IL-36α, IL-36β and IL-36γ production are illustrated in the microphotographs for non-cancer (**b**, **e**, and **h**) and cancer tissues (**c f** and **i**). ****: *P* < 0.0001. The bar represents 30 μm

Colonic IL-36α, IL-36β and IL-36γ were detected in the non-cancer colon samples, mainly localised in the cytoplasm of colonic epithelial cells and goblet cells (Fig. 1b, e and h, respectively). In contrast, there was much weaker colonic staining for IL-36α, IL-36β and IL-36γ in the CRC tissues (Fig. 1c, f and i, respectively) with a diffuse distribution in the poorly differentiated cancer cells. Quantitative analysis demonstrated that IL-36α, IL-36β or IL-36γ were reduced by 60, 80% or 70% in the CRC tissues, compared to that of patient-matched non-cancer colonic tissue, respectively (*P* < 0.001) (Fig. 1a, d, g).

### The ROC curves and survival curves associated with IL-36α, IL-36β and IL-36γ production in CRC patients

**Fig. 2:**
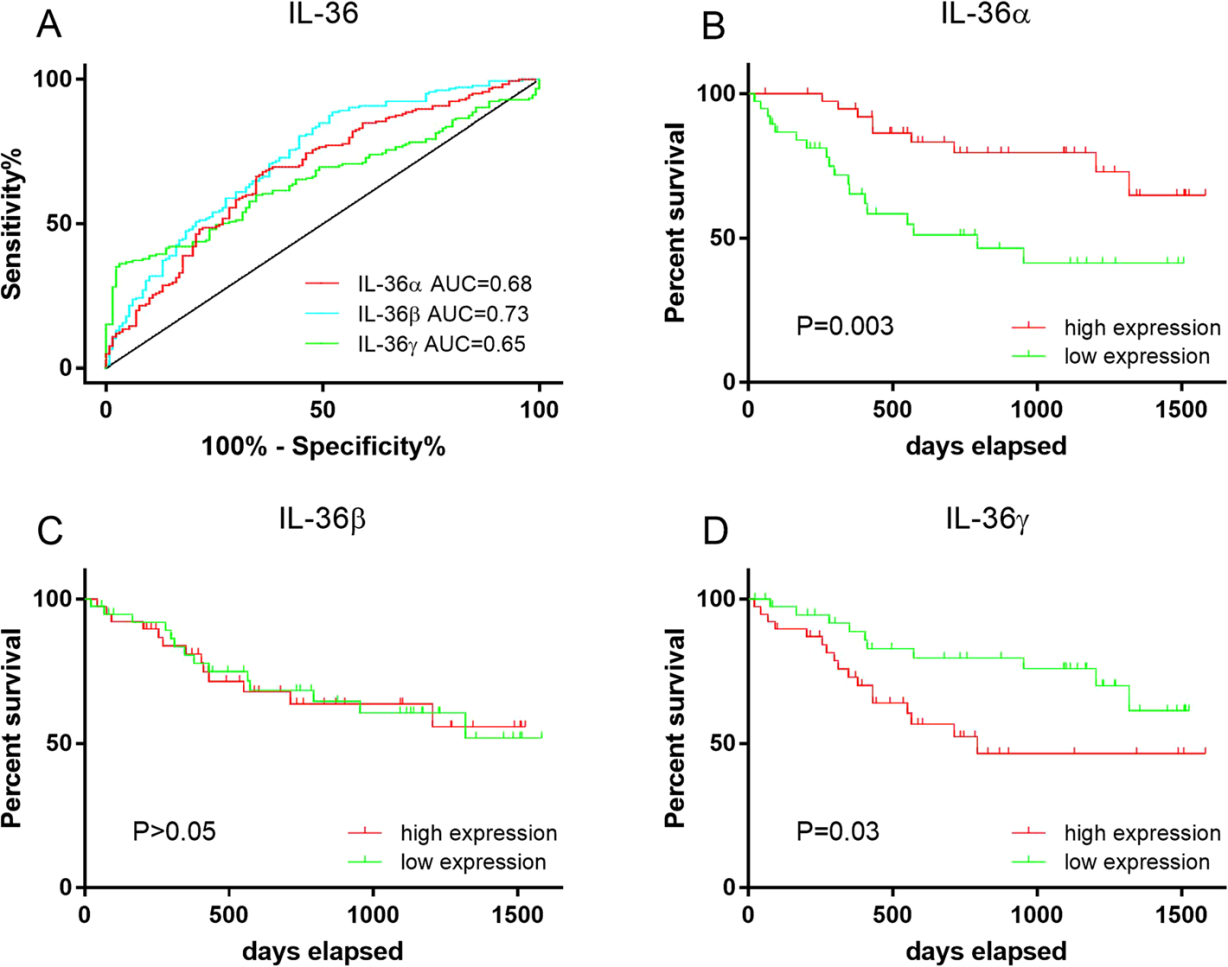
The ROC curve and survival curve analysis of the production of IL-36α, IL-36β and IL-36γ among the CRC patients. The specificity versus sensitivity of IL-36α, IL-36β and IL-36γ (**a**) in CRC is shown using ROC curves (**a**). Area under the curve, IL-36α: AUC = 0.68; IL-36β: AUC = 0.73; IL-36γ: AUC = 0.65. Survival curves comparing high and low levels of production of IL-36α (**b**), IL-36β (**c**) and IL-36γ (**d**) are shown, using the Kaplan-Meier method and the log-rank test

ROC curve analysis was applied to determine the specificity and sensitivity of IL-36α, IL-36β and IL-36γ production for prediction of CRC outcomes in both CRC and non-cancer tissues. The area under the curve (AUC) of the ROC curves drawn for IL-36α, IL-36β and IL-36γ production were 0.68, 0.73 and 0.65, respectively (Fig. 2a), suggesting that IL-36β is slightly better than IL-36α or IL-36γ in terms of specificity and sensitivity for the detection of CRC.

Based on the level of production of each of the IL-36 s, patients were stratified into either high or low production groups, using the median of IL-36 s production. Kaplan-Meier survival curves for each of the IL-36 s were then generated by log-rank test and sub-group analysis was used to determine the relationship between various clinicopathological characteristics and the capacity of IL-36 s production to predict survival.

Our data demonstrate that the IL-36α^high^ group had a significantly longer survival than IL-36α^low^ CRC patients (*P* = 0.003, Fig. 2b). Although there was a difference in colonic IL-36β production in the CRC compared to non-cancer control, there was no significant difference in overall survival period of the CRC patients between IL-36β^high^ and IL-36β^low^ (*P* > 0.05, Fig. 2c). As for IL-36γ, our data demonstrate that the survival among those CRC patients from the IL-36γ^low^ group was significantly longer than those CRC patients from the IL-36γ^high^ group (*P* = 0.03, Fig. 2d).

### Association between combinations of IL-36α, IL-36β or IL-36γ production level and survival curves in CRC patients

**Fig. 3:**
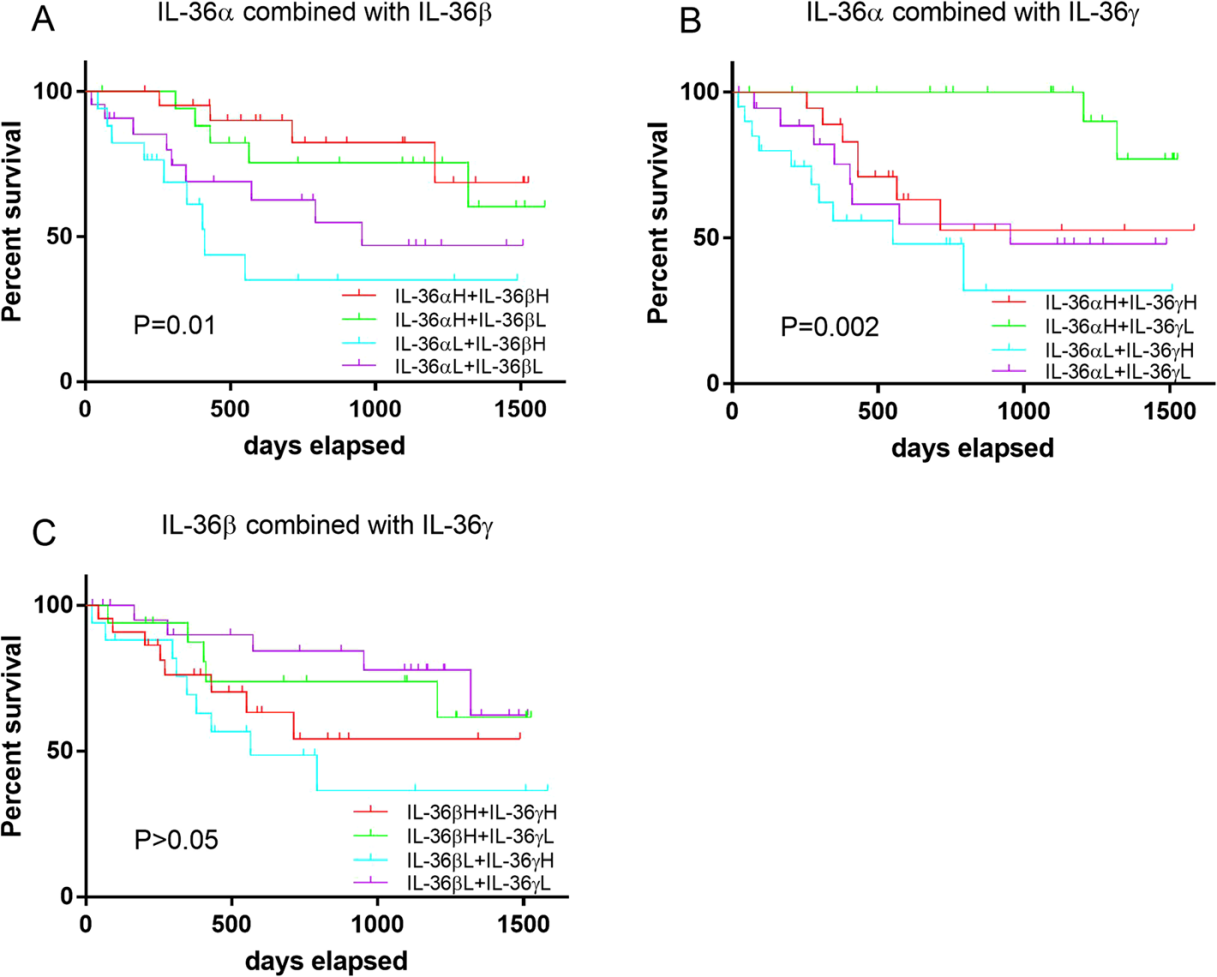
Survival curve analysis for sub-groups of CRC patients when defined by combined IL-36α and IL-36β production level (**a**) or IL-36α and IL-36γ (**b**), or IL-36β and IL-36γ (**c**), using the Kaplan-Meier method and the log-rank test

Since the level of production of IL-36β did not significantly predict survival of patients, patients were further stratified into four groups based on the level of production if IL-36α plus IL-36γ and the survival of these patient groups was analysed (Fig. 3b). The longest survival was observed among the CRC patients with IL-36α^high^ plus IL-36γ^low^ production post-surgery; whereas the shortest survival was detected in the CRC patients with IL-36α^low^ plus IL-36γ^high^ production (*P* = 0.002, Fig. 3b). To consider a possible synergistic effect from a combination of IL-36β with either IL-36α or IL-36γ, survival curves were generated for these sub-groups (Fig. 3a and c). The longest survival amongst these groups was observed in the CRC patients with IL-36α^high^ plus IL-36β^high^, while the shortest survival was in the CRC patients with IL-36α^low^ plus IL-36β^high^ (*P* = 0.01, Fig. 3a). However, there was no significant difference of survival rate among the CRC patients when subgroups were stratified based on IL-36β plus IL-36γ production (*P* > 0.05, Fig. 3c).

### Survival in CRC patients within sub-groups based on IL-36α and IL-36γ production further stratified according to clinicopathological parameters

**Fig. 4:**
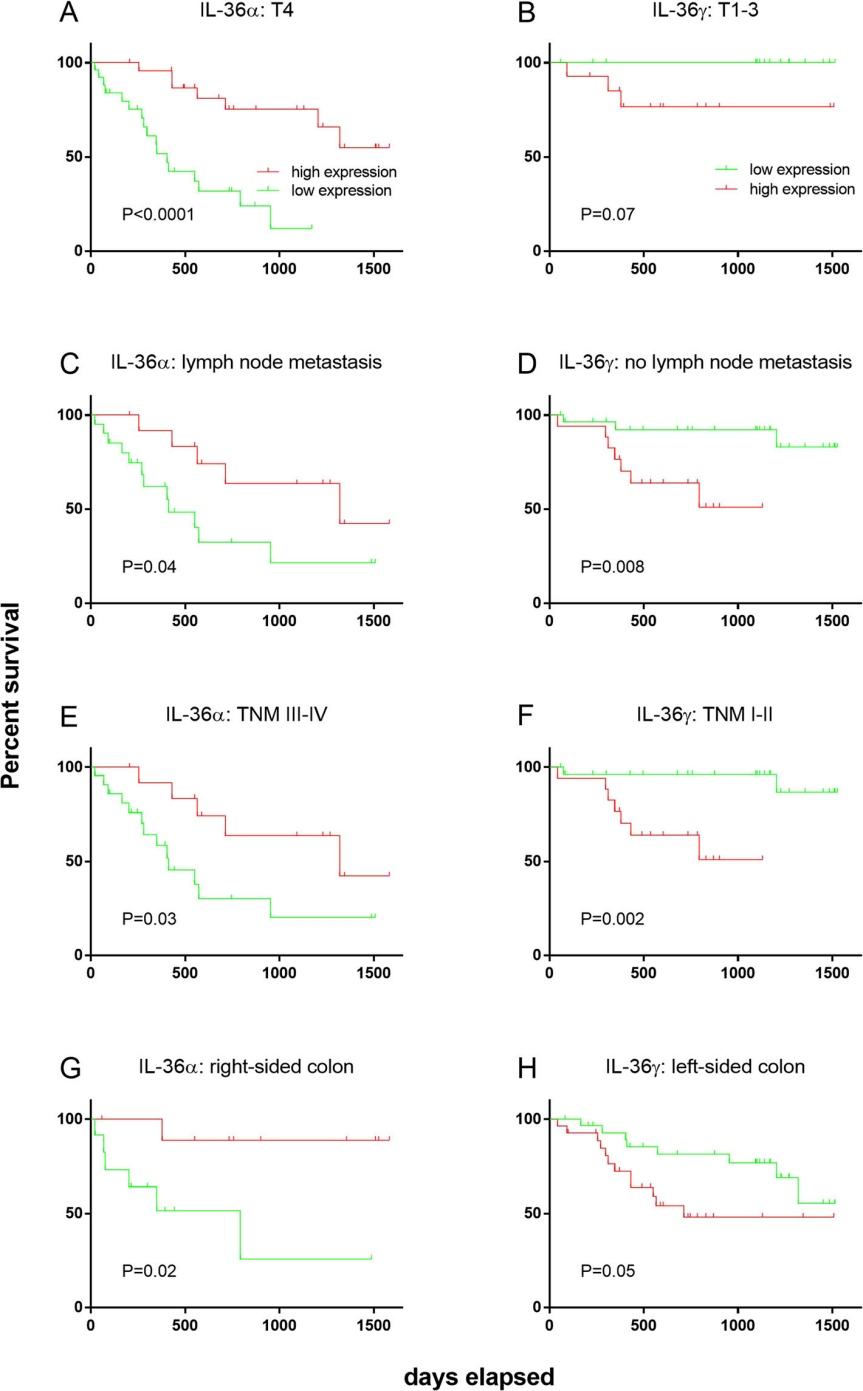
Survival curve analysis for sub-groups of CRC patients when defined by clinical presentation at surgery, comparing high and low expression levels of IL-36 s: by IL-36α production in patients with an invasion depth of T4 (**a**), lymph node metastasis (**c**), TNM III-IV (**e**), or right-sided colon CRC (**g**); or by IL-36γ production in patients with an invasion depth of T1–3 (**b**), no lymph node metastasis (**d**), TNM I-II (**f**), or left-sided colon CRC (**h**), using the Kaplan-Meier method and the log-rank test. X-axis represents days elapsed

Patients sub-groups stratified by IL-36 s production were further stratified according to clinicopathological parameters and survival within these sub-groups was analysed using the Kaplan-Meier method and the log-rank test. Significant differences between survival curves were observed between IL-36α^high^ and IL-36α^low^ CRC patients only in those patients with an invasion depth of T4 (*P* < 0.0001, Fig. 4a), the presence of lymph node metastasis (*P* = 0.04, Fig. 4c), in patients who were TNM III-IV (*P* = 0.03, Fig. 4e), and in patients with a right-sided CRC (*P* = 0.02, Fig. 4g). In all cases, IL-36α^high^ patients exhibited improved survival.

The application of the same analysis using the Kaplan-Meier method and the log-rank test to IL-36γ, detected significant differences in the survival curves of CRC patients between IL-36γ^high^ and IL-36γ^low^ patients only in patients with no lymph node metastasis (*P* = 0.008, Fig. 4d), in patients who were TNM I-II (*P* = 0.002, Fig. 4f), and in patients with a left-sided CRC (*P* = 0.05, Fig. 4h). There was no significant difference in the survival curves of CRC patients where the invasion depth was T1–3 (*P* = 0.07, Fig. 4b), although a trend was observed. In all cases, IL-36γ^low^ patients exhibited improved survival.

However, there was no significant difference in survival curves between IL-36α^high^ and IL-36α^low^ CRC patients with an invasion depth of T1–3 (*P* = 0.8, SFig 1A), patients with no lymph node metastasis (*P* = 0.08, SFig 1C), patients who were TNM I-II (*P* = 0.1, SFig 1E), and patients with a left-sided CRC (*P* = 0.06, SFig 1G), using the Kaplan-Meier method and the log-rank test. There was also no significant difference for survival curves between IL-36γ^high^ and IL-36γ^low^ in patients with an invasion depth of T4 (P = 0.1, SFig 1B), patients with lymph node metastasis (P = 0.8, SFig 1D), patients who were TNM III-IV (*P* = 0.7, SFig 1F), and patients with a right-sided CRC (*P* = 0.5, SFig 1H).

### Univariate and multivariate analyses to determine the relationship between survival of CRC patients and IL-36α, IL-36β and IL-36γ production

The following variables were considered in the multivariate analysis: IL-36α, β, γ, age, lymph node metastasis, depth of invasion, TNM. These variables were selected because they were significant in univariate analysis, or because they became significant in multivariate analysis, or were primary variables (i.e. the three IL-36 s) (Table 2).

**Table 2:** Univariate and multivariate analysis of IL-36 s and clinicopathological factors affecting survival of patients with CRC

Characteristics	No. Pt	Univariate analysis		Multivariate analysis	
		HR (95% CI)	*P* value	HR (95% CI)	*P* value
IL-36α		0.31 (0.13–0.69)	0.004	0.37 (0.16–0.87)	0.02
High	39/79				
Low	40/79				
IL-36β		1.02 (0.48–2.18)	NS		
High	39/79				
Low	40/79				
IL-36γ		2.33 (1.05–5.16)	0.04	2.18 (0.95–5.04)	NS
High	39/79				
Low	40/79				
Gender		1.02 (0.45–2.27)	NS		
Male	52/79				
Female	27/79				
Age (years)		1.70 (0.78–3.70)	NS	2.46 (1.04–5.83)	0.04
< 70	38/79				
≥70	41/79				
Position		1.09 (0.46–2.60)	NS		
Right-sided	22/79				
Left-sided	57/79				
Size (diameter, cm)		1.84 (0.80–4.21)	NS		
≤5	63/79				
> 5	16/79				
Lymph node metastasis		2.87 (1.31–6.28)	0.008	0.78 (0.16–3.72)	NS
No	45/79				
Yes	34/79				
Differentiation		1.78 (0.71–4.48)	NS		
Well	1/79				
Moderate	66/79				
Poor	12/79				
Invasion depth		3.30 (1.38–7.87)	0.007	3.47 (1.34–8.99)	0.01
T1	2/79				
T2	14/79				
T3	13/79				
T4	50/79				
TNM		2.47 (1.46–4.18)	0.001	2.54 (0.74–8.69)	NS
I	12/79				
II	32/79				
III	32/79				
IV	3/79				

*HR* hazard ratio, *CI* confidence interval, *P* values for Cox proportional hazards regression analysis

Univariate analysis was applied to determine the contributions of the factors analysed above to the prediction of survival rate. Univariate and multivariate analyses were selected for determining CRC survival rate, as described previously [25].

IL-36α (HR, 0.31; 95%CI, 0.13–0.69; *P* = 0.004), IL-36γ (HR, 2.33; 95%CI, 1.05–5.16; *P* = 0.04), lymph node metastasis (HR, 2.87; 95%CI, 1.31–6.28; *P* = 0.008), tumour invasion depth (HR, 3.30; 95%CI, 1.38–7.87; *P* = 0.007) and TNM (HR, 2.47; 95%CI, 1.46–4.18; *P* = 0.001) were found to be good predictors for prognosis in univariate analyses for survival of patients with CRC. Interestingly, only IL-36α (HR, 0.37; 95%CI, 0.16–0.87; *P* = 0.02), age (HR, 2.46; 95%CI, 1.04–5.83; P = 0.04) and tumour invasion depth (HR, 3.47; 95%CI, 1.34–8.99; *P* = 0.01) were found to be independent and reliable biomarkers in multivariate analysis for predicting survival rate among these CRC patients (Table 2). However, other factors, including IL-36β, IL-36γ, sex, position, size, lymph node metastasis, differentiation and TNM were not significant in multivariate analysis among these CRC patients.

## Discussion

In the current study we demonstrated that colonic IL-36α, IL-36β and IL-36γ were substantially reduced in CRC compared to that of the paired non-CRC tissues. The sensitivity versus specificity of IL-36α, IL-36β and IL-36γ production were determined using ROC curve analysis [28] and were found to be predictive of the presence of cancer, yielding AUC values of 0.68, 0.73 and 0.65 for IL-36α, IL-36β or IL-36γ, respectively. Although these values were statistically significant, they are not sufficiently high to be used as reliable biomarkers for colorectal cancer.

Survival rate is an objective indicator for evaluating postoperative CRC prognosis [29]. Based on our current data, IL-36α^high^ CRC patients have a better survival than IL-36α^low^ CRC patients. Additionally, IL-36α is an independent factor affecting the survival of CRC patients on multi-variate analysis. This is supported by the finding that IL-36α may exhibit anti-tumour effects in CRC progression [13], perhaps via activating adaptive T cell immune responses and recruiting CD3^+^ and CD8^+^ tumour infiltrating lymphocytes (TILs) [30].

Our survival data from multivariate analysis for IL-36α production is supported by Wang et al. [13]. However, Wang et al.*,* determined colonic IL-36α production only in CRC tissue from CRC patients, without using adjacent non-cancer tissue as a control. In addition, the classification of IL-36α as either high or low was based on a naked eye scoring system. On the other hand, our quantification was performed objectively using computerised software (ImagePro Plus 9.1), which is routinely used in our research group [21], in addition to the comparison with non-cancer paired colonic tissues. Thus, our rigorous data are probably more convincing and reliable.

Additionally, we observed that CRC patients within the colonic IL-36γ^low^ production group had a better survival rate than IL-36γ^high^. It has been reported that IL-36γ is mainly produced by M1 macrophages in the CRC tumour micro-environment [14], which may contribute to tumoricidal effects [31]. Our speculation is that disturbance of differentiation of macrophages may be involve in the development of CRC, which will be clarified in future investigations. We speculate that there are differential regulatory roles of IL-36 during the development of CRC. Our hypothesis is supported by others, showing the distinct expressions of IL-36α, IL-36β, and IL-36γ, and their antagonist (IL-36Ra) in autoimmune disease in human and animal models [32, 33]. The precise underlying mechanism of these differential roles of IL-36 s remains to be explored in our future experiment.

On the other hand, surprisingly, there was no statistically significant difference in survival rate between IL-36β^high^ and IL-36β^low^ production groups in CRC patients, suggesting that IL-36β may not be a good marker for predicting prognosis of CRC. However, our observation that colonic mucosal IL-36β was ~ 80% reduced in CRC tissue suggests that IL-36β may participate in inhibiting the development of CRC. Interestingly, IL-36α and IL-36γ, but not IL-36β, are upregulated at the molecular and cellular levels in inflammatory bowel disease (IBD) [34], although IL-36α, IL-36β and IL-36γ are all pro-inflammatory cytokines [11]. This observation from Nishida et al. suggests that only IL-36α and IL-36γ, but not IL-36β, contribute to the pathogenesis of intestinal inflammation. The discrepancy in IL-36β production between Nishida, in intestinal inflammation where little change was observed, and our current findings in CRC where a substantial reduction in IL-36β was observed, may be due to the different severity of the diseases and/or different pathogenesis. Nevertheless, based on the observation from Nishida et al. and ourselves, we speculate that, to contribute to pathogenesis, the production of IL-36β may require substantial alteration, i.e. a moderate to minor change in IL-36β in IBD, but a substantial change in CRC. The precise mechanism of IL-36β in oncogenesis, particularly in the development of CRC, will be further investigated, particularly its signalling pathways.

Interestingly, a stratification into sub-groups using a combination of IL-36α plus IL-36γ production provided a better prognostic outcome than when IL-36α or IL-36γ production only was evaluated in CRC patients. Specifically, a larger and more distinct difference was observed between the sub-group containing IL-36α^high^ plus IL-36γ^low^ patients compared to the sub-group containing IL-36α^low^ plus IL-36γ^high^ patients, when evaluated using survival curves. Such an observation might be useful in clinical decision making in the management of CRC patients.

It has been reported that 4.5 cm is the optimal cut-off value for the whole colon, receiver-operator characteristic (ROC) analysis has been applied to different parts of the large bowel, and has determined the following cut-off values of 5 cm, 5.3 cm, 3.9 cm, and 3.4 cm have the strongest discriminatory capacity for the whole colon, right-sided colon, left-sided colon, and rectal cancers, respectively [35]. Thus, a 5 cm cut-off is a good size to utilise. We will, of course, use such defined location sizing for our future studies.

We are wary that the different cut offs that have been chosen may influence the statistical significance of the results. Thus, the interpretation of our current study should be cautious. More highly powered studies should serve to clarify this potential issue.

A recent paper on TNM staging in CRC questioned the validity of full TNM staging as a prognostic indicator, rather suggesting that simple evaluation of mesenteric node spread was more highly predictive. Thus, the TNM system is quite controversial [36]. Thus, we have elected to use both simple nodal involvement and full TNM to circumvent this controversy and also to improve our statistical correlation. Notably, both nodal involvement and TNM score were strongly statistically correlated with IL36α expression by univariate analysis, but not by multivariate analysis. While the referee is correct in stating that T contributes to TNM, TNM contains considerable additional contributions to its score, that potentially could mask a correlation with IL-36 s expression. Indeed, we observed that only T remained significant in multivariate analysis.

The interval between malignant transformation at the cellular level and tissue diagnosis is difficult to determine and may vary depending on the pathogenic pathway underpinning the CRC (microsatellite instability/mismatch repair gene mutations versus Wnt/APC pathway) [15, 16]. The extent to which pathway pathogenesis influences the development of variation in IL-36 s expression is therefore difficult to formulate. We elected to exclude mucinous adenocarcinoma CRC samples typically associated with mismatch repair gene mutations to minimise this potential factor.

Furthermore, we observed that IL-36α and IL-36γ were good indicators for prognosis for sub-groups of CRC patients when stratified by clinicopathological characteristics, particularly when comparing the more advanced stages of CRC to earlier stages. The TNM staging system incorporates the depth of invasion and lymph node metastasis of tumours, which are known to be two of the influential factors determining prognosis of CRC patients [37] [36]. Although there was no significant difference between IL-36γ^high^ and IL-36γ^low^ in patients that were T1–3, there was a trend, which may be due to the relatively small number of followed-up patients, especially the T1–3 patients, who generally had survived well, with very few deaths. We also acknowledge that the speculative role of IL-36α, β or γ in predicting survival based on different T status, TNM staging, LN metastasis and right vs left side cancer in the current study. However as stated above, the precise differential regulatory role of IL-36 s in the carcinogenesis is being investigated in genetically manipulated animals.

Finally, there is often both a difference in pathogenesis and stage at diagnosis between left-sided and right-sided CRC, in part due to different clinical presentation and treatment [38]. This is consistent with our finding that the CRC patients with right-sided colon CRC, versus left-sided colon CRC, mostly presented at a late stage by TNM [39].

## Conclusion

We found that the level of IL-36α or IL-36γ production in CRC seems to be reliable biomarkers in predicting the prognosis of CRC at the later or early stage of CRC, respectively. Combining an assessment of IL-36α plus IL-36γ production can more accurately determine the postoperative prognosis of CRC patients. Furthermore, our data may be useful in the development of the new approaches to the management of CRC.

## Data Availability

Yes

## Abbreviations

AUC: Area under the curve
CRC: Colorectal cancer
DCs: Dendritic cells
IL: Interleukin
MAPKs: Mitogen-activated protein kinase
NFkb: Nuclear factor kappa B
ROC: Receiver operating characteristic
TNM: Tumour, node and metastasis
